# Implication of inter-joint coordination on the limb symmetry index measured during the seated single-arm horizontal push test

**DOI:** 10.3389/fspor.2025.1531366

**Published:** 2025-02-10

**Authors:** Y. Blache, M. Degot, T. de Sousa, I. Rogowski

**Affiliations:** Université Lyon 1, LIBM – UR 7424, Villeurbanne, France

**Keywords:** unilateral seated shot-put test, shoulder, bilateral asymmetry, power, functional performance test

## Abstract

**Introduction:**

The seated single-arm horizontal push test (SSAHPT) could be used to assess unilateral upper-extremity power. While superior performance of the dominant side compared to the nondominant one (LSI) is often observed, causes of this bilateral imbalance remain unclear. This study aimed to assess the influence of upper-extremity dominance on both inter-joint coordination and joint contribution in SSAHPT.

**Methods:**

Twenty-five healthy male athletes were fitted with reflective markers and performed SSAHPT with the dominant and nondominant sides. Humerothoracic, elbow and wrist joint contributions to the horizontal medicine ball velocity were computed. The temporal occurrence of joint peak contribution was used to assess inter-joint coordination.

**Results:**

The temporal occurrence of joint peak contribution occurred in a proximo-to-distal sequence at the dominant side, while at the nondominant side, joint peak contribution first occurred at shoulder, then simultaneously at elbow and wrist. The elbow joint contributed the most to the horizontal medicine ball velocity, but its relative contribution was significantly greater for the nondominant limb than the dominant one (*p* < 0.05).

**Discussion:**

These findings highlight that SSAHPT bilateral asymmetry is explained by a change in motor patterns, as inter-joint coordination and contribution, between the dominant and nondominant sides. From a practical perspective, our findings suggest that for healthy athletes, firstly the LSI observed during SSAHPT may not be used as a good indicator of bilateral imbalance in upper-extremity power, and secondly SSAHPT performance reflects primarily elbow joint velocity capacities and then shoulder ones.

## 1 Introduction

The unilateral seated shot-put test (USSPT), also named the seated single-arm shot-put test (1), was initially designed to assess the explosive power of the upper-extremities by measuring the maximal distance achieved when putting an overweight ball (2). Over the past decade, several modifications have emerged, regarding the seated position (1, 3) and medicine ball mass (1). Recently, Degot et al. (4), described a procedure involving a seated single-arm horizontal push test (SSAHPT) to avoid bilateral differences in release angle. Nevertheless, regardless of the experimental procedure employed, it has consistently been observed that in healthy individuals, the distance achieved when using the dominant side is between 3% and 11% greater than that achieved with the nondominant side (1–4). The medicine-ball pushing task has therefore been advocated to be an appropriate easy-to-use test for assessing bilateral imbalance in upper-extremity power.

The limb symmetry index (LSI) can be used to depict the bilateral imbalance between the dominant and nondominant limbs (4). For USSPT, the LSI has initially been described as a valid indicator of bilateral power imbalance (1). Similar height and angle at medicine ball release for the dominant and nondominant sides were used to advocate that the coordination demand of the upper-extremity during USSPT was lower in comparison to throwing tests, hence reflecting upper-extremity power independently of coordination (1). However, more recent studies revealed a systematic bias between USSPT LSI and LSI calculated from shoulder and elbow strength assessed isokinetically. Indeed, unlike USSPT, no side-to-side differences in shoulder and elbow (5) or pushing (6) isokinetic strength were detected. Such a bias appears crucial in practical terms, as it suggests that the LSI observed in USSPT may ultimately not be used as a true indicator for characterizing bilateral upper-extremity power imbalance. Furthermore, a prior study pointed out that during overarm throwing, differences in inter-joint coordination between the dominant and nondominant limbs may account for the observed decline in ball velocity in the nondominant limb (7). Drawing an analogy to overarm throwing, one might assume that the LSI observed during medicine ball push, particularly in SSAHPT, results from bilateral differences in inter-joint coordination. However, to our knowledge, no study has yet investigated this hypothesis. Therefore, the understanding of the underpinning factors of the LSI and the potential involvement of inter-joint coordination in medicine ball pushing tasks still demands to be investigated.

The distance achieved by the medicine ball in the USSPT provides a broad overview of each upper-extremity power, but falls short in explaining the underlying factors contributing to the bilateral asymmetry commonly observed in this task. The construct validity of the USSPT performance to upper-extremity strength revealed positive relationships between USSPT performance and upper-extremity pushing strength (6). USSPT performance also exhibited strong positive correlations with isokinetic strength measured for internal and external glenohumeral rotation (8), shoulder flexion (5) and elbow extension (5). By comparing the strength of the relationship between USSPT performance and elbow or shoulder joint isokinetic strength Watson et al. (5) concluded that for both sides, each joint contributed equally to USSPT performance. However, bivariate correlation analysis alone is insufficient for truly determining the *in situ* contribution of each upper-extremity joint to medicine ball push performance. Moreover, the aforementioned studies focused solely on the shoulder and elbow joints, overlooking the potential role of the wrist, while a prior study (9) has highlighted its positive contribution to shot put performance. Concerning SSAHPT specifically, no study has yet elucidated the individual joint contribution to medicine ball distance achieved. To gain a deeper understanding of the underlying factors driving medicine ball pushing tasks performance, it is then necessary to specifically examine the contributions of each joint in-situ.

To address the gap in the literature regarding the role of upper-extremity inter-joint coordination and the individual joint contribution to SSAHPT performance, our study aimed to examine the influence of upper-extremity dominance on both inter-joint coordination and joint contribution in SSAHPT. In line with the literature showing alteration of inter-joint coordination for the nondominant upper-extremity in ball throwing (7), it was first hypothesized that inter-joint coordination was affected for the nondominant upper-extremity. Second, considering the equal relationship between elbow or shoulder joint isokinetic strength and medicine ball push performance (5), it was hypothesis that these two joints contributed equally to SSAHPT performance regardless of the upper-extremity side. Such findings would help coaches and clinicians firstly to determine if the LSI observed during SSAHPT is related to a deficit of upper-extremity power or an alteration in inter-joint coordination, and secondly to elucidate whether the performance achieved during SSAHPT depends more on shoulder or elbow functional capacities.

## 2 Materials and methods

### 2.1 Participants

A convenient sample composed of 25 healthy male athletes (age: 23.7 ± 3.0 yrs; mass: 73.6 ± 8.4 kg; height: 179.0 ± 6.5 cm; weekly specific sport practice: 5.4 ± 2.3 h; weekly physical training: 2.3 ± 2.8 h; sport practice: climbing (5), fighting sports (4), workout (3), handball (3), kayak (2), swimming (2), basketball (1), volleyball (1), soccer goalkeeper (1), rugby (1), gymnastic (1) and multi-sport(1)) was included in this study, which was approved by the local ethical committee of the University of Lyon (CER-UdL #2022-10-13-002). All participants signed an informed consent. Inclusion criteria were to be aged from 18–35 years, practice a physical activity involving upper-extremities at least 2 h per week. Exclusion criteria were to declare any injury at the shoulder or the upper-extremity for the 6 months preceding the evaluation.

### 2.2 Procedure and data collection

Prior to the test, each participant performed a standardized warm up (10), which consisted in performing with a 2 kg medicine ball, 10 elbow flexion-extension, 10 humeral flexion-extension, 10 push-pull, 10 waist-revolutions, 10 head-revolutions; followed by 5 pushes-up against wall with wide base hand placement and 5 with narrow base hand placement, 15-s right-lateral-core, 30-s frontal-core and 15- left-lateral-core training (see Supplementary File for more details). Then they were fitted with spherical reflective markers located on the thorax (*n* = 6) and bilaterally on the humerus (*n* = 7), forearm (*n* = 3) and hand (*n* = 4). Four markers were also secured on a 3 kg medicine ball. For further calibration of the kinematic model, the participant held a static pose for five seconds. Finally, the procedure described by Degot et al. (4) was followed. After one submaximal and one maximal trial of familiarization, the participant performed three maximal SSAHPT with the dominant and nondominant sides, randomly. Briefly, the participant sat on the floor with knees bent at about 90°, feet flat, and half of the back kept in contact with a vertical support. The participant held the 3 kg medicine ball at shoulder height while flexing the elbow, and then pushed it as far as possible in a horizontal forward direction. It was asked to the participant to keep their head, scapula of the non-test side, and back in contact with the vertical support and his nonthrowing arm in his belly. A 30-s recovery period was set between each SSAHPT. Each trial was visually controlled to avoid any deviations from pose instructions, any countermovement or parabolic path. In such cases, the trial was cancelled, and a new maximal push was performed. Raw marker trajectories were recorded using a 14-cameras optoelectronic system at 200 Hz (Qualysis, Sweden).

### 2.3 Data treatment

Raw coordinates of the markers were filtered using a low-pass Butterworth filter (4th order) with a 10 Hz cut-off frequency. The pushing motion begun when the horizontal coordinate of the medicine ball increased, and ended when the horizontal velocity of the medicine ball became constant. This last event defined the medicine ball release, at which the release angle was computed relative to the horizontal plane with respect to the world axis origin, and the release height corresponded to the vertical distance from the ground to the medicine ball center. SSAHPT distance, namely the horizontal flying distance of the medicine ball from release to touch-down, was estimated based on projectile mechanics law (see Supplementary File for more details). SSAHPT distance was first computed in meters, and then normalized allometrically, i.e., in m/kg^0.35^ (2). To avoid any bilateral differences in release heights or angles for further analysis, only the best trial per side presenting differences with the contralateral side inferior to 2° for release angle and to 4 cm for release height were kept for analysis. These cutoffs were chosen as they involved less than 5% variability in SSAHPT distance (unpublished data).

For each side, unilateral participant-specific kinematic model was calibrated from the static posture by following the International Society of Biomechanics recommendations (11). The kinematic model was composed of six segments, namely the ground, thorax, humerus, forearm, hand and medicine ball. It was actuated by 6 degrees of freedom between the ground and thorax, 6 degrees of freedom between the thorax and the humerus (Humerothoracic joint), 2 rotations between the humerus and forearm (Elbow joint), 3 rotations between the forearm and the hand (Wrist joint) and 3 translations between the hand and medicine ball center. Joint angles were computed using a multibody kinematic optimization consisting of minimizing the Euclidian distance between experimental markers and virtual markers of the kinematic model (12). The following sequences of rotation were chosen: medio-lateral inclination, antero-posterior bending and internal-external rotation for the thorax relative to the ground; abduction-adduction, internal-external rotation and flexion-extension for the humerothoracic joint; flexion-extension and prono-supination for the elbow joint; flexion-extension, radio-ulnar deviation and internal-external rotation for the wrist joint.

The horizontal velocity of the medicine ball center over the time was computed as the matrix product of joint angular velocities by the jacobian matrix (i.e., partial derivative of medicine ball coordinates relative to joint angles). The decomposition of the matrix product provided the absolute contribution of each joint to the horizontal medicine ball velocity (see Supplementary File for more details). Relative joint contribution to the horizontal medicine ball velocity was obtained by dividing the absolute contribution by the horizontal velocity at release instant. Inter-joint coordination was characterized by computing the instant when the peak of each absolute joint contribution occurred. These instants were normalized by the movement duration. A proximo-to-distal inter-joint coordination consisted of peak occurring firstly for the shoulder, secondly for the elbow and finally for the wrist.

### 2.4 Statistical analysis

After having checked the normality of the differences with a shapiro-wilk test, paired t-test were used to compare SSAHPT distances, release angles and heights between the dominant and nondominant sides. Cohen'd effect sizes were computed and interpreted as weak, medium and large for 0.2, 0.5 and 0.8, respectively.

To characterize inter-joint coordination, non-parametric Friedman's analyses of variance (i.e., one per side) were used to assess the influence of the joint (Humerothoracic vs. Elbow vs. Wrist) on the instants of peak joint contribution occurrence. Kendall's W effect sizes were computed and interpreted as small, moderate and large for <0.3, [0.3–0.5[ and ≥0.5, respectively. In the case of significant effect, Wilcoxon tests were performed for pairwise comparisons with Bonferroni correction. r effect sizes were computed and interpreted as small, moderate and large for [0.1–0.3[, [0.3–0.5[ and ≥0.5, respectively.

The effects of *joint* (Humerothoracic vs. Elbow vs. Wrist) and *dominance* (Dominant vs. Nondominant) factors and their interaction on mean absolute and relative joint contributions to medicine ball horizontal velocity was assessed using linear mixed models. Participants were entered as random intercept. *η*² effect sizes were computed and interpreted as weak, medium and large for 0.01, 0.06 and >0.14, respectively. Linearity and homoscedasticity of residuals were graphically controlled. When significant effects were found, Tuckey *post-hoc* tests were applied.

All analyses were made using RStudio 2022.07.2 (RStudio Team, Boston, United-States) and the significant level was set at *p* ≤ 0.05.

## 3 Results

SSAHPT distances were on average 6.4 ± 6.7% [CI95 = 2.6%] higher for the dominant side in comparison to the nondominant side, while no significant differences were observed for release angle and height (Table 1).

**Table 1:** Mean ± standard deviation [95% confidence interval] SSAHPT distance, release angle and height for the dominant (Dom) and nondominant (NDom) sides.

	Dom	NDom	*p*	ES	Power
SSAHPT distance (cm/kg^0.35^)	87.1 ± 8.2 [1.4]	81.4 ± 8.4 [1.3]	<0.001	0.66	0.89
Release angle (°)	3.5 ± 3.6 [0.03]	3.3 ± 3.2 [0.03]	0.63	0.07	0.06
Release height (cm)	92.4 ± 6.5 [3.2]	94.5 ± 5.8 [3.3]	0.21	0.34	0.37

ES for effect size.

**Figure 1:**
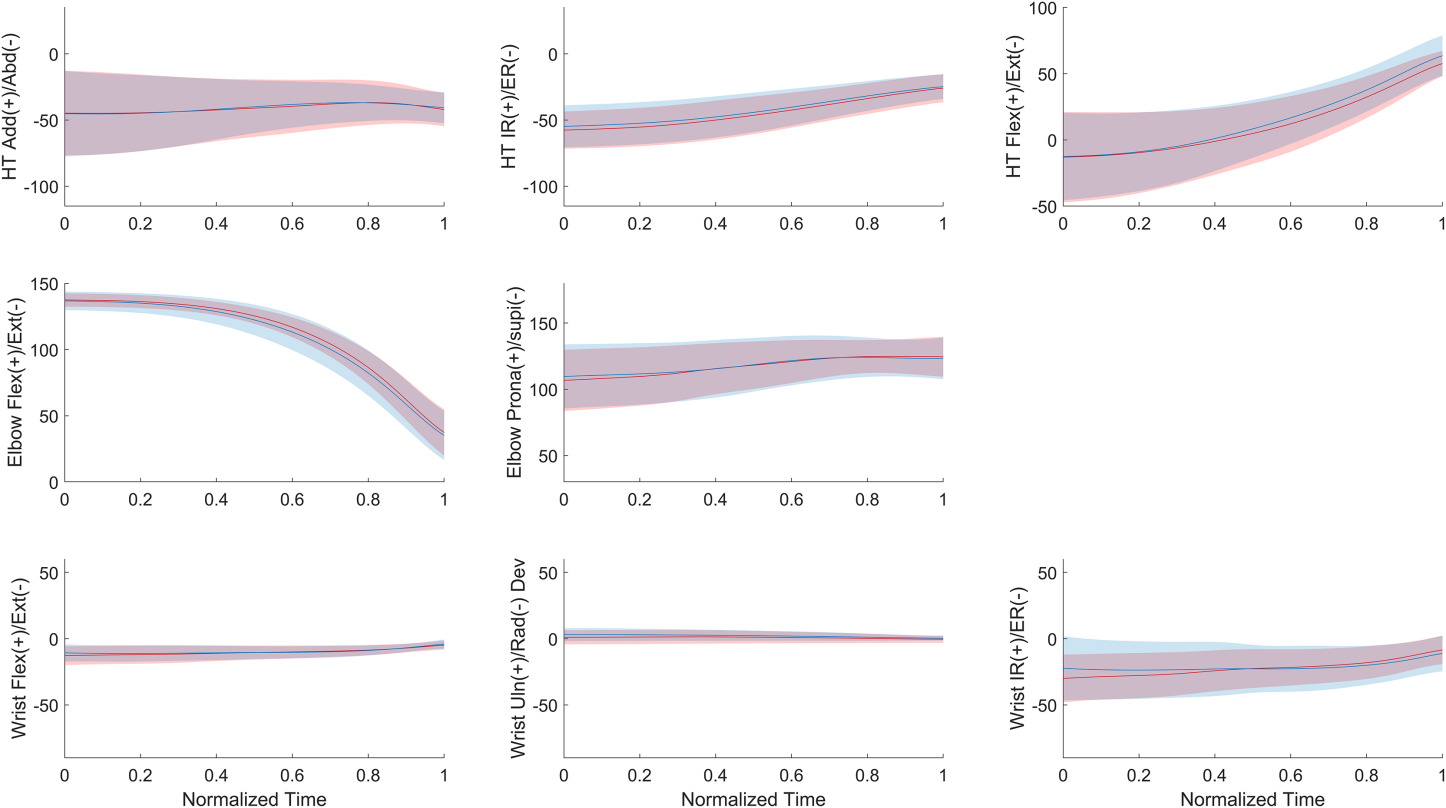
Mean (± standard deviation) joint angles with respect to the normalized time (0 for the beginning of the movement and 1 for medicine ball release instant) for the dominant (red) and nondominant (blue) sides. HT for humerothoracic, IR and ER for internal and external rotation, Add and Abd for adduction and abduction respectively, Flex and Ext for flexion and extension respectively, Prona and Supi for pronation and supination respectively, Uln and Rad Dev for ulnar and radial deviation respectivey.

The multibody kinematic optimization revealed that, for both the sides, medicine ball pushing was mainly achieved by humerothoracic flexion [Range of Motion (RoM) ≈ 50°], humerothoracic internal rotation (RoM ≈ 30°) and elbow extension (RoM ≈ 100°), while other rotations remained stable throughout the entire movement (RoM < 10°) (Figure 1).

**Figure 2:**
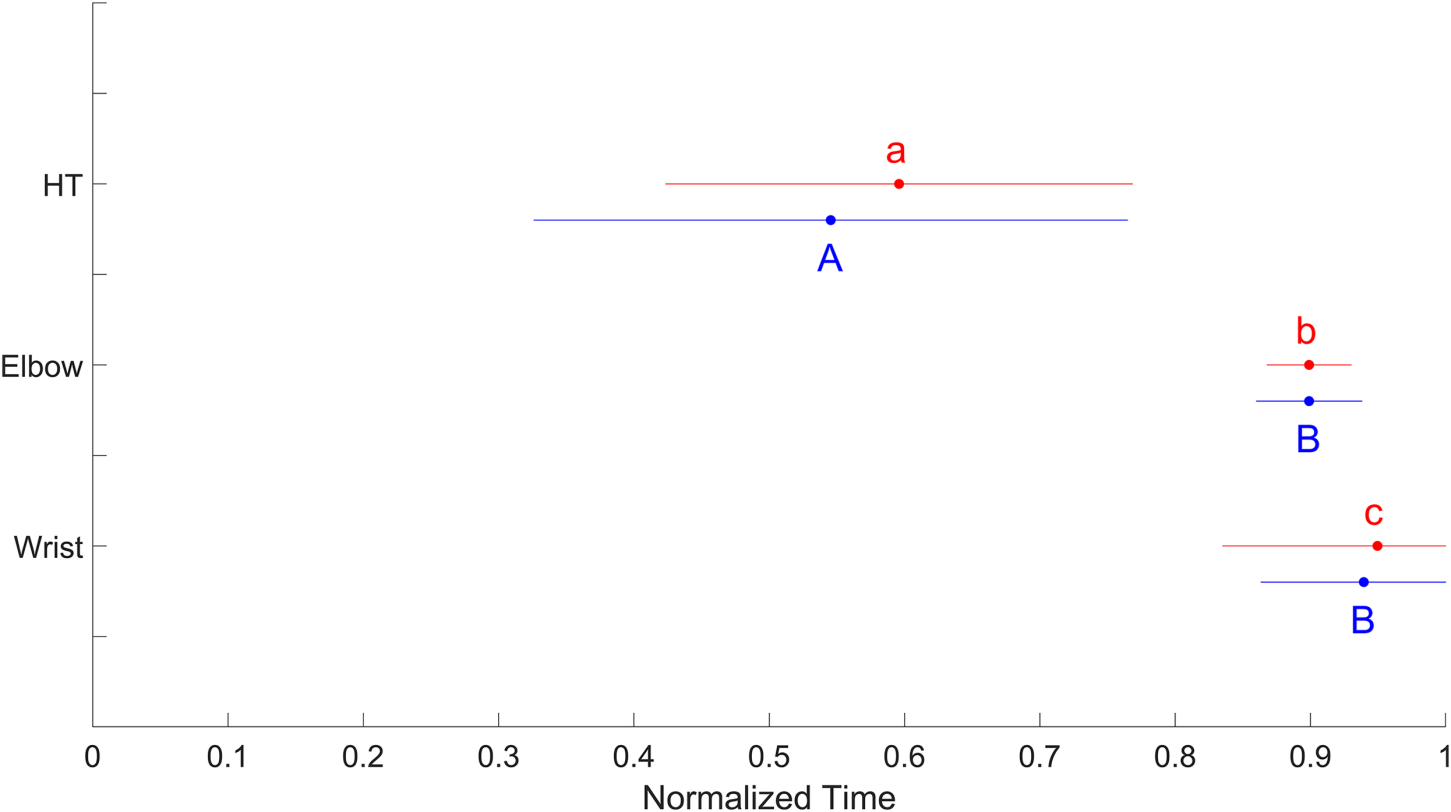
Mean (± standard deviation) normalized time of peak contribution for the humerothoracic (HT), elbow and wrist joints of the dominant (red) and nondominant (blue) sides. For each side, means that do not share the same letter are significantly different (*p* < 0.01).

For the dominant side, Friedman's analysis of variance (*W* = 0.78, large effect, *p* < 0.001) followed by Wilcoxon tests revealed that the peak contribution for the humerothoracic joint occurred significantly earlier than that for the elbow joint (*r* = 0.86, large effect, *p* < 0.01, power = 0.98), which occurred significantly earlier than that for the wrist joint (*r* = 0.60, large effect, *p* < 0.01, power = 0.80). For the nondominant side, Friedman's analysis of variance (*W* = 0.81, large effect, *p* < 0.001) followed by Wilcoxon tests showed that the peak contribution of humerothoracic joint firstly occurred (*r* = 0.87, large effect, *p* < 0.001, power = 0.98), followed simultaneously by those of the elbow and wrist joints (*r* = 0.43, moderate effect, *p* = 0.14, power = 0.52) (Figure 2).

**Figure 3:**
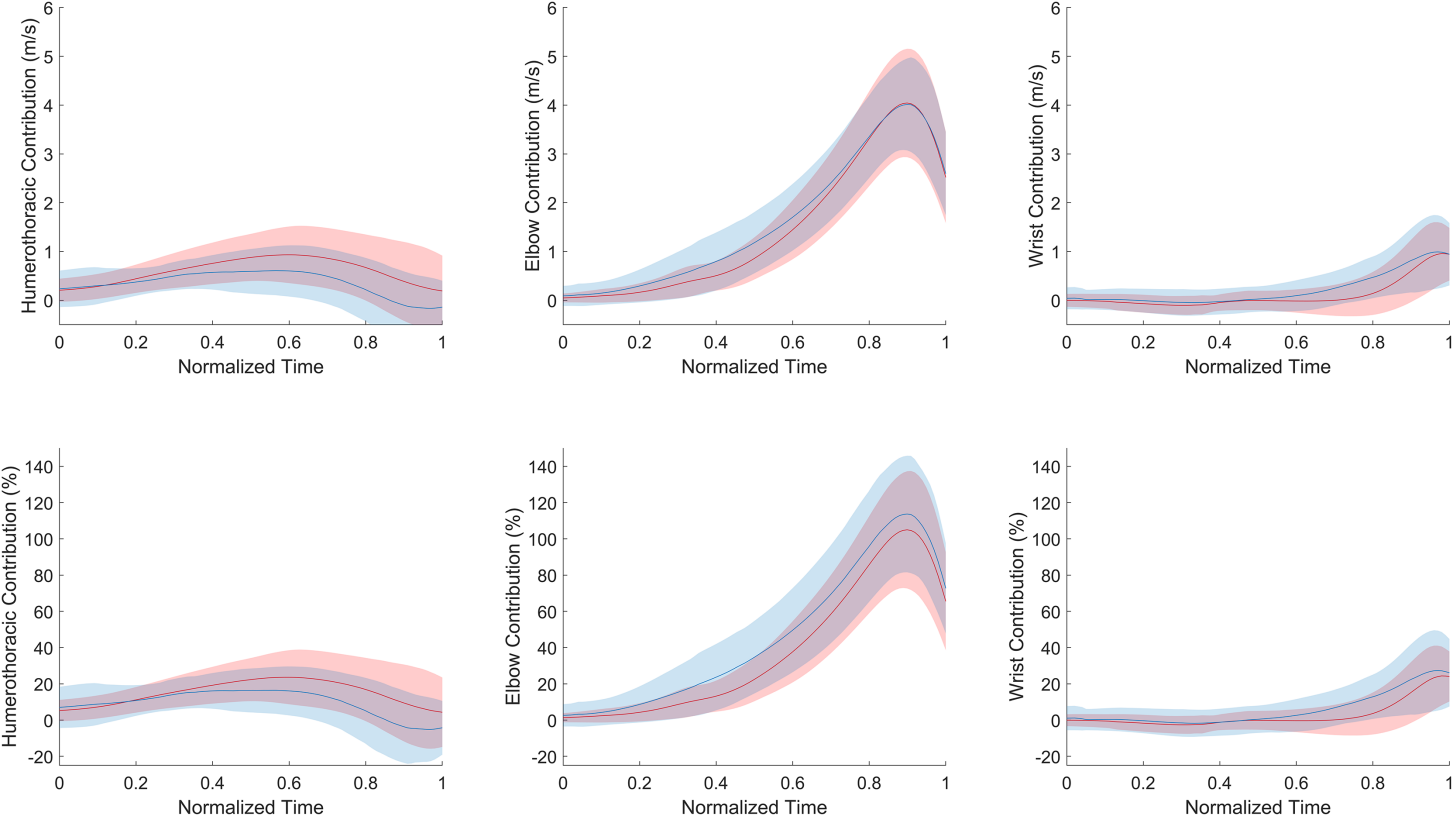
Mean (± standard deviation) absolute (top) and relative (bottom) joint contribution to medicine ball horizontal velocity with respect to the normalized time (0 for the beginning of the movement and 1 for medicine ball release instant) for the dominant (red) and nondominant (blue) sides. HT for humerothoracic.

Linear mixed models revealed a significant interaction effect joint*dominance on mean joint absolute contributions [*η*² = 0.08, medium effect, F_(2, 115)_ = 5.07, *p* = 0.008]. Elbow joint presented the highest absolute contribution to medicine ball horizontal velocity for both the sides (*p* < 0.0001). Humerothoracic joint had a significant greater absolute contribution than the wrist joint for the dominant side (*p* < 0.0001), while humerothoracic and wrist joints had similar absolute contribution for the nondominant side (*p* = 0.40). Mean absolute humerothoracic contribution to medicine ball horizontal velocity was about 1.7 time greater for the dominant side in comparison to the nondominant one, while no significant differences between sides were observed for the elbow and wrist joints (Figure 3 and Table 2).

**Table 2:** Mean ± standard deviation [95% confidence interval] absolute (m.s^−1^) and relative (%) joint contribution to the medicine ball horizontal velocity for the dominant (Dom) and nondominant (NDom) sides.

		Dom	NDom	*p*	ES	Power
Absolute	Humerothoracic	**0.60** **±** **0.39** [0.15]*	**0.36** **±** **0.30** [0.12]*	**0**.**04**	**0**.**55**	**0**.**75**
	Elbow	1.45 ± 0.41 [0.16]	1.60 ± 0.43 [0.17]	0.40	0.24	0.21
	Wrist	0.10 ± 0.17 [0.07]*^,^**	0.21 ± 0.20 [0.08]*	0.40	0.42	0.52
Relative	Humerothoracic	15.1 ± 9.7 [3.8]*	9.7 ± 8.2 [3.2]*	0.19	0.46	0.60
	Elbow	**37.8** **±** **12.0** [4.7]	**45.9** **±** **15.9** [6.2]	**0**.**03**	**0**.**44**	**0**.**56**
	Wrist	2.6 ± 4.5 [1.8]*^,^**	5.7 ± 5.6 [2.2]*	0.36	0.44	0.56

ES for effect size; is written in bold for a significant difference between the Dominant and nondominant sides.

* means significantly different from Elbow joint contribution.

** means significantly different from Humerothoracic joint contribution.

Linear mixed models also revealed a significant interaction effect joint*dominance on mean joint relative contributions [*η*² = 0.09, medium effect, F_(2, 115)_ = 5.59, *p* = 0.005]. Elbow joint presented the highest relative contribution for both sides (*p* < 0.0001). Relative humerothoracic contribution was significantly higher compared to wrist joint for the dominant side (*p* < 0.001), while no difference was observed between these two joints for the nondominant side (Figure 3 and Table 2). Mean relative elbow contribution to medicine ball horizontal velocity was 8.2 ± 18.8% greater for the nondominant side in comparison to the dominant one, while no significant differences between sides were observed for the humerothoracic and wrist joints (Figure 3 and Table 2).

## 4 Discussion

The present study aimed to assess the influence of upper-extremity dominance on both inter-joint coordination and joint contribution in SSAHPT. Our findings confirmed the first hypothesis as the proximo-to-distal sequence in peak joint contribution observed for the dominant limb was altered for the nondominant one. By contrast the second hypothesis was not supported since for both sides the elbow joint contributed more than the humerothoracic joint to SSAHPT performance. From a practical perspective, these findings suggest that for healthy athletes, firstly the LSI observed during SSAHPT may not be used as a good indicator of bilateral imbalance in upper-extremity power, and secondly SSAHPT performance reflects primarily elbow joint velocity capacities and then shoulder ones.

The velocity of the end-effector in a kinematic chain, i.e., the hand or the ball in throwing tasks, is influenced by two factors: the torque generated by each joint and the velocity-dependent torques (13). The latter refer to the indirect effects of one joint torque on the angular velocities of all other joints (14). When attempting to maximize the speed of the medicine ball, the accelerations of the proximal joints, like the trunk and shoulder, are mainly related to their own generated joint torques. In contrast, the accelerations of the distal segments are primarily a result of the velocity-dependent torques (14). For instance, the flexion joint torque of the shoulder during a medicine ball throw can indirectly generate an extension velocity of the elbow joint without any action of elbow extensors muscles. This concept underlies the proximo-to-distal joint coordination observed in various sport activities, such as overarm throwing (7), shot-put (15) and punching (16). This coordination pattern was observed in our cohort when SSAHPT was performed with the dominant upper-extremity. However, when the SSAHPT was achieved with the nondominant upper-extremity, the proximo-to-distal sequence was disrupted, especially between the elbow and wrist joints, potentially affecting the performance of the nondominant side (17). These inter-limb differences may be explained by the dynamic-dominance hypothesis (18) stating that the dominant arm presents an advantage in exploiting interaction torques in comparison to the nondominant-one. Our results highlight then that the decreased performance for the nondominant side may be partly due to a lack of desynchronization in joint excursion at the distal upper extremity, leading to less efficient inter-joint coordination. In consequence, these findings outcomes challenge the use of SSAHPT LSI to reflect upper-extremity power imbalance.

Based on the comparison with isokinetic assessment, Watson et al. (5) concluded that USSPT performance was equally explained by the humerothoracic and elbow joints. Our findings were different for the SSAHPT since, when measured *in situ*, the contribution of the elbow joint exceeded that of the humerothoracic joint. These controversial findings between Watson et al. (5) results and ours can likely be attributed to the differences in methodologies (bivariate correlation analyses vs. in-motion analysis) and procedures (USSPT vs. SSAHPT), but also by the presence of humerothoracic internal rotation during the SSAHPT (Figure 1), which is not accounted for in isokinetic assessments of shoulder flexion strength (19). Hence, the observed involvement of humerothoracic internal rotation in our study may substantiate previously established relationships between isokinetic strength assessment for internal glenohumeral rotation and USSPT performance (8). On the other hand, while the wrist contributed to the SSAHPT performance, its influence was relatively minor compared to the proximal joints for the dominant side, except during the latter part of the movement (Figure 3), confirming that wrist contribution should not be totally overlooked during pushing tasks (9). Furthermore, the reduced SSAHPT performance observed in the nondominant limb was primarily explained by a decrease in absolute humerothoracic joint contribution. As a result, when considering the relative contributions to the horizontal velocity of the medicine ball, the elbow joint plays a more significant role in SSAHPT performance when using the nondominant upper-extremity compared to the dominant one. These findings first support an heterogenous contribution of upper-extremity joints to SSAHPT performance, and second that a change in motor patterns was noted between the dominant and nondominant sides.

From a practical perspective, our findings suggest that, for healthy athletes, the LSI observed during SSAHPT may not be used as a good indicator of bilateral imbalance in upper-extremity power. Indeed, a bilateral difference in SSAHPT performance may result mainly from an alteration in inter-joint coordination and not power deficit (6). Therefore, coaches or clinicians, should analyze the performance achieved by each upper-extremity individually without performing bilateral comparison, and the contralateral limb might not be used as a reference to estimate upper-extremity power impairment through the SSAHPT. Coaches and clinicians might consider combining SSAHPT with complementary analytical tests (e.g., shoulder flexion and internal rotation, and elbow extension strength assessment) to determine whether rehabilitation should focus on power deficit and/or inter-joint coordination deficits. Additionally, even if SSAHPT is a multi-joint upper-extremity functional performance test (5), our findings suggest that the performance achieved is mainly explained by the elbow and then the shoulder joint. In consequence, when coaches or clinicians use SSAHPT for assessing indirectly upper-extremity power, they should notice that they primarily assess elbow joint velocity capacities and secondly shoulder ones. Finally, using solely the performance achieved during SSAHPT may be insufficient for coaches and clinicians to truly understand physical capacities of their athletes since inter-joint coordination may be altered and heterogenous joint contribution explains the performance. In consequence, the development of markerless motion capture systems using a smartphone camera (20) appears to be a promising approach for real-time assessment of inter-joint coordination and joint contribution in athletes during medicine-ball pushing task, and then assisting coaches and clinicians in their diagnoses.

The current study is not without limitations. First, although our observed LSI falls within the previously reported range (i.e., between 3% and 11%), we cannot affirm that our findings are applicable to other single arm shot procedures described in the literature (1–3). Indeed, differences in seated position (halfback vs. fullback support) and medicine ball push constraints (horizontal vs. free path) limit the applicability of our results to the SSAHPT. Second, our study used a kinematic approach to assess inter-joint coordination and joint contribution to SSAHPT performance, while a muscular approach through electromyography or musculoskeletal modeling would provide a deeper understanding of the biomechanical factors involved in SSAHPT. Finally, one notable limitation of this study is its inability to be generalized to healthy female athletes, on the one hand, and, on the other hand, different outcomes may arise among athletes with prior injuries. Future investigations should focus on the underlying components explaining bilateral imbalance in medicine-ball pushing task performance among athletes who have experienced upper-extremity injuries.

## 5 Conclusions

This study evidences that inter-joint coordination assessed through peak joint velocity contributions follows a proximo-to-distal sequence for the dominant limb during SSAHPT, while this sequence is altered when using the nondominant limb. Although a nonuniform joint contribution to the horizontal medicine ball velocity is observed in favor of the elbow joint for both the sides, the relative contribution of the later is the highest for the nondominant upper-extremity. These findings support the notion that SSAHPT LSI is explained by a change in motor patterns between the dominant and nondominant sides. From a practical perspective, our findings suggest that for healthy athletes, firstly the LSI observed during SSAHPT may not be used as a good indicator of bilateral imbalance in upper-extremity power, and secondly SSAHPT performance reflects primarily elbow joint velocity capacities and then shoulder ones.

## Data Availability

The raw data supporting the conclusions of this article will be made available by the authors, without undue reservation.
