# Supplementary material for: Implication of inter-joint coordination on the limb symmetry index measured during the seated single-arm horizontal push test
Source: Front Sports Act Living. 2025 Feb 10;7:1531366. doi: 10.3389/fspor.2025.1531366 (PMC11847852; doi:10.3389/fspor.2025.1531366)
Supplement: Supplementary file 1 [file Datasheet1.pdf]

Supplementary Material

1 Supplementary Data

Warm up description

10 elbow flexion-extension with a 2-kg medicine ball	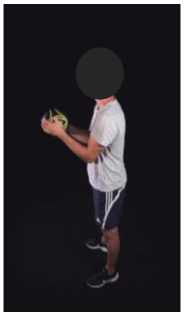	10 humeral flexion-extension with a 2-kg medicine ball	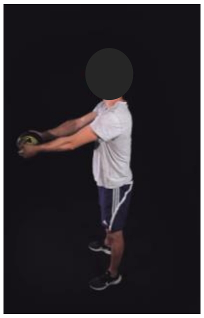
10 push-pull with a 2-kg medicine ball	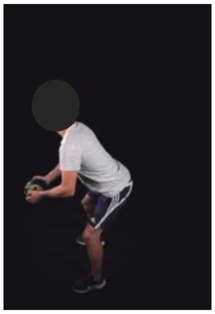	10 waist-revolutions with a 2-kg medicine ball	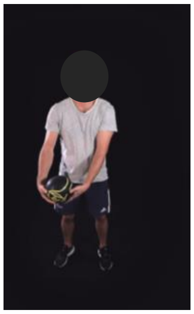
10 head-revolutions with a 2-kg medicine ball	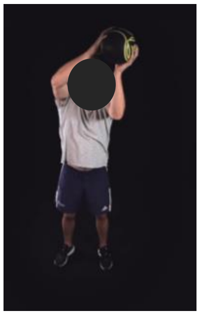	5 pushes-up against wall with wide base hand placement	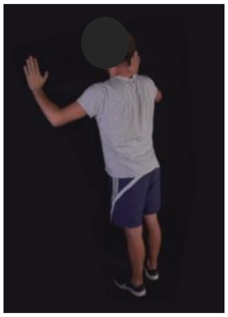

5 pushes-up against wall with narrow base hand placement	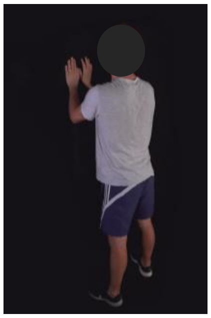	15-s right-lateral-core training	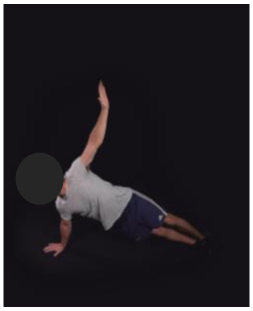
30-s frontal-core training	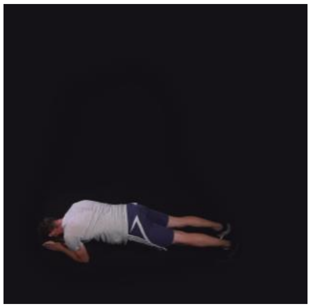	15- left-lateral-core training	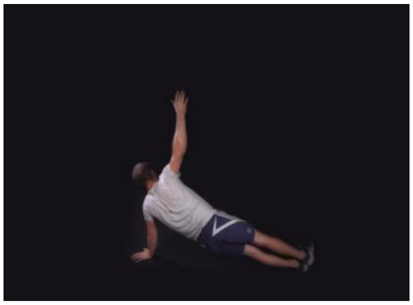

Procedure to compute medicine ball horizontal distance from projectile mechanical law

The projectile height (y) during the flight phase is defined by Equation 1

$$y = \frac{1}{2} \cdot g_y \cdot t^2 + \sin\alpha \cdot V_0 \cdot t + y_0\ , \qquad \text{(Eq.1)}$$

with $g_y = -9.81\ m.s^{-2}$, t: the flying time, α, V_0, y_0: projectile release angle, velocity and height respectively

From Eq.1, when the projectile touch the ground (end of the flying phase):

$$0 = \frac{1}{2} \cdot g_y \cdot t^2 + \sin\alpha \cdot V_0 \cdot t + y_0 \qquad \text{(Eq.2)}$$

When solving Equation 2, we obtain the total flight time $\left(t_{flight}\right)$

$$t_{flight} = \frac{-\sin\alpha \cdot V_0 + \sqrt{\sin\alpha^2 \cdot V_0^2 - 2 \cdot g_y \cdot y_0}}{g_y} \qquad \text{(Eq.3)}$$

Finally, projectile horizontal distance (H_{dist}) is obtained with Equation 4

$$H_{dist} = \cos\alpha\ \cdot V_0 \cdot t_{flight} \qquad \text{(Eq.4)}$$

This figure represents the medicine ball trajectory from release instant (time = 0 s) measured with video cameras (blue line) and reconstructed from projectile mechanical law (red dot line).

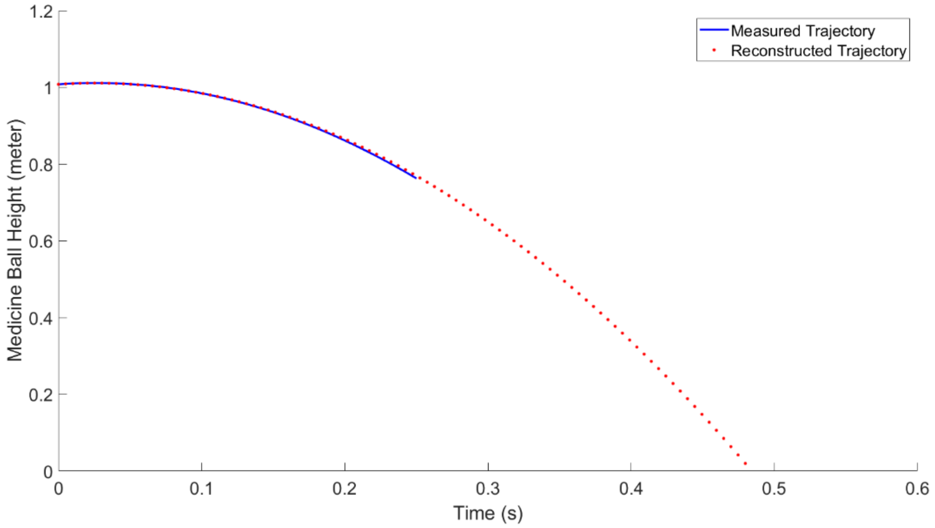

Procedure to determine joint contribution to medicine ball velocity

The kinematic chain is composed of four segments: Thorax, Arm, Forearm and Wrist.

Joints (q) between segments are modeled as follow:

- Humerothoracic joint (Thorax-Arm): 3 degrees of freedom (3 rotations)
- Elbow joint (Arm-Forearm): 2 degrees of freedom (2 rotations)
- Wrist joint (Foream-hand): 3 degrees of freedom (3 rotations)

Medicine ball velocity ($\dot{MB}$) with respect to the time (t) is computed with Eq. 5:

$$\dot{MB}(t) = \sum_{i=1}^{i=8} J_{q_i}(t) \,.\, \dot{q}_i(t) \qquad \text{(Eq. 5)}$$

with i the upper-extremity degrees of freedom (Humerothoracic [n=3], Elbow [n=2] and Wrist [n=3]), $\dot{q}_i$ the joint angular velocity for each degree of freedom, J the jacobian matrix (i.e. the partial derivative of the medicine ball coordinates (MB) relative to joint angles).

$$J_{q_i} = \left[\frac{\partial MB}{\partial q_1} \quad \cdots \quad \frac{\partial MB}{\partial q_8}\right],$$

The decomposition of Eq. 1 is:

$$\dot{M}B(t) = \begin{bmatrix} J_{q_1}(t).\dot{q}_1(t) + \\ J_{q_2}(t).\dot{q}_2(t) + \\ J_{q_3}(t).\dot{q}_3(t) + \\ J_{q_4}(t).\dot{q}_4(t) + \\ J_{q_5}(t).\dot{q}_5(t) + \\ J_{q_6}(t).\dot{q}_6(t) + \\ J_{q_7}(t).\dot{q}_7(t) + \\ J_{q_8}(t).\dot{q}_8(t) \end{bmatrix},$$

with the absolute contribution of the humerothoracic joint (sum from 1 to 3), elbow joint (sum from 4 to 5) and wrist joint (sum from 6 to 8) to the medicine ball velocity.
